# The prevalence and clustering of metabolic syndrome risk components in Chinese population: a cross-sectional study

**DOI:** 10.3389/fendo.2023.1290855

**Published:** 2023-12-13

**Authors:** Xu Zhao, Cihang Lu, Bo Song, Deshi Chen, Di Teng, Zhongyan Shan, Weiping Teng

**Affiliations:** The Department of Endocrinology and Metabolism, Institute of Endocrinology, National Health Commission Key Laboratory of Diagnosis and Treatment of Thyroid Diseases, The First Hospital of China Medical University, Shenyang, Liaoning, China

**Keywords:** metabolic syndrome, obesity, prevalence, components, tide

## Abstract

**Objective:**

The metabolic syndrome (MetS) is diagnosed upon the manifestation of ≥ 3 out of 5 specific components. The present study evaluated the epidemiological characteristics of the MetS components and their clustering condition among Chinese adults.

**Methods:**

68383 participants aged 18-80 years from TIDE were scored on a six-point (0–5) MetS severity score (MSSS), which quantified their cumulative amount of MetS risk components. We evaluated the epidemiological characteristics of these components and their clustering conditions. Additionally, we examined the relation of age with the prevalence of different MSSSs or specific MetS components using restricted cubic splines.

**Results:**

Among 68383 participants, 26113 men and 24582 women had abnormal MetS components. There were significant differences in most epidemiological characteristics between the 6 MSSS groups. The top three prevalence of abnormal metabolic components were high systolic blood pressure (SBP) (9.41%, n=6568), high waist circumference (WC) (8.13%, n=6120), and the cooccurrence of high SBP and high WC (6.33%, n=4622). Participants were more likely to have all five MetS components when HDL-C was low. Restricted cubic splines showed that when the MSSS ≥3, the MetS prevalence of male peaked and that of the female population increased most rapidly at 40-60 age group.

**Conclusion:**

The 40-60 age group can be regarded as the high-risk period of MetS, and elderly women have a higher risk of multiple metabolic disorders than men. The top three clustering of abnormal metabolic components were high SBP, high WC, and their combination. Multiple components aggregation was more likely to occur when HDL-C decreased.

## 1 Introduction

Metabolic syndrome (MetS) has become a global problem. MetS is considered a useful clinical construct of clustered metabolic risk factors that are common in the general population (1). The principal components for MetS include obesity, dyslipidemia (low high-density lipoprotein and/or elevated triglycerides), elevated blood pressure, and alterations in glucose metabolism. It can lead to a greater risk of type-2 diabetes, lipid disorders, cardiovascular disease, hepatic steatosis, and other circulatory disorders (2). It likely derives from overnutrition, often combined with a sedentary lifestyle (3).

The authors of the National Health and Nutrition Examination Survey (NHANES) concluded that the prevalence of MetS significantly increased between 1999 and 2014 (27.9% to 31.5%) (4), and NHANES data from 2011 to 2016 showed that the weighted MetS prevalence was 34.7% (95% CI, 33.1%-36.3% [n=5885]) among 17048 participants (5). According to a global survey of obesity in 195 countries performed in 2015, 604 million adults and 108 million children were with obesity (6). According to CDC data published in 2017, approximately 12.2% of adults in the USA had type-2 diabetes (T2DM) (7). According to the International Diabetes Federation (IDF) diabetes atlas (8), the global prevalence of diabetes was 8.8% (415 m) as of 2015 and is expected to increase to 10.4% (642 m) by 2040. Since MetS is approximately three times more common than diabetes, the global prevalence can be estimated to be approximately one-quarter of the world’s population (9). The China Health and Retirement Longitudinal Study (CHARLS) clarified that the estimated prevalence of MetS in 2015 was 20.41% (95% CI: 19.02-21.8%) (10). China Nutrition and Health Surveillance (2015-2017) data showed that the standardized prevalence of high waist circumference, high blood pressure and low high-density lipoprotein cholesterol were 40.8%, 49.4% and 41.1%, respectively (11). The standardized prevalence of MetS among Chinese residents aged 20 years or older was 31.1%, with a significantly higher prevalence in women than in men (32.3% vs. 30.0%). Moreover, MetS, not its components, was significantly associated with all-cause mortality (12).

The prevalence of metabolic disorders varies with gender, sex, race/ethnicity, and heredity, along with lifestyle. Understanding which factors are associated with a high MetS rate would help to identify individuals who are at a greater risk of MetS. Data from the 2011 and 2015 waves of CHARLS showed that a higher prevalence of MetS is found in those who reported being middle aged or elderly, being female, and residing in northern China or living in urban areas (10). Additionally, the MetS Severity Score (MSSS) quantifies the cumulative amount of risk derived from the presence of MetS risk factors (13). Another nationwide cross-sectional survey reported that Korean ethnicity was associated with a higher prevalence of the five components of MetS (14). Moreover, the rapid increase in MetS prevalence has raised many public health concerns, including concerns regarding cancer, such as colorectal and breast cancers (15). Not only the metabolic syndrome itself, but also some studies have shown that the aggregation of more components or the presence of a particular component is associated with certain diseases or adverse outcomes. Sung Keun Park found that the presence of ≥4 metabolic components of MetS leads to a higher risk of pancreatic cancer (16). Also, research has found that MetS is closely related to the poor prognosis of endometrial cancer (EC) patients. As the prognosis of EC patients worsens, the prevalence of MetS components increases (17). Given that the prevalence of MetS and its individual components (particularly obesity and insulin resistance) has increased significantly in recent decades, greater efforts are needed to reduce the incidence of this condition and its components. In addition, certain components may be more strongly linked to MetS (18). Nevertheless, the characteristics of the MetS components and their aggregation numbers have not been systematically reported. The present study evaluated the epidemiological characteristics of the components of MetS and their clustering condition among Chinese residents aged 18-80 years based on data from the Thyroid Disorders, Iodine Status and Diabetes Epidemiology (TIDE) study, a national epidemiological cross-sectional study, which was conducted from 2015 to 2017 and covered all 31 provinces of mainland China. Through this study, we can be more targeted to the prevention and treatment of Mets and related components.

## 2 Materials and methods

### 2.1 Study population

**Figure 1:**
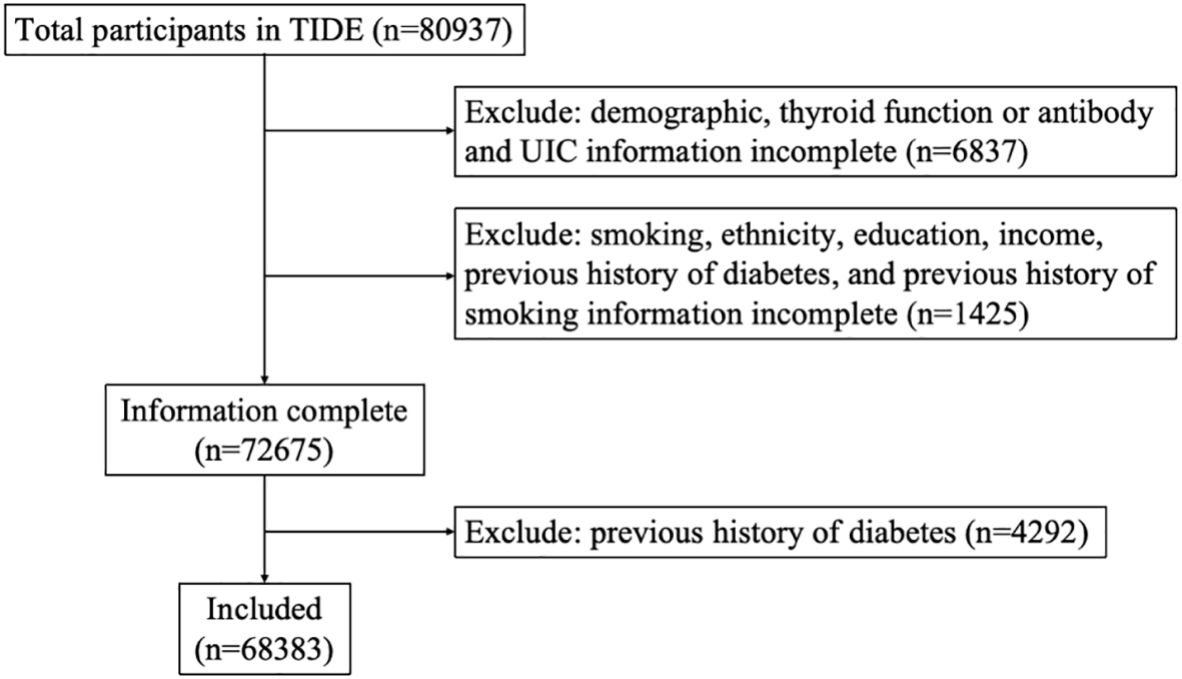
Flowchart of participants’ inclusion and exclusion criteria. TIDE, Thyroid Disorders, Iodine Status and Diabetes, a national epidemiological cross-sectional study.

TIDE utilized a multistage stratified sampling method based on the latest 2010 national census data. According to the population size and economic level, one representative city was selected from each province in 31 provinces of China. Eligible respondents were randomly selected from residential areas. The sampling method was the same in rural areas as in urban areas. Specific sampling and implementation methods have been published previously (19) and are described in **Supplementary Methods**. The inclusion criteria were as follows: aged 18-80 years old; lived in the local area for at least five years; no use of iodine-containing drugs or contrast media within 3 months of participating in the study; and nonpregnant women. A total of 80937 people participated in the program. Each participant provided written informed consent before data collection. This study followed the 2013 revised Declaration of Helsinki, and the procedures followed were approved by the Ethics Committee of China Medical University (2014-103-2; serial number: IRB [2008]115). Exclusion criteria: missing demographic data (ethnicity, education, income), and missing data on smoking status, blood glycosylated hemoglobin, blood lipids, body mass index (BMI), waist circumference, and previous history of diabetes, thyroid function and antibodies, urinary iodine. On this basis, people with a history of diabetes, hypertension, and cardiovascular disease were excluded. Finally, a total of 68383 subjects were included in this study. The flow chart of patient inclusion is shown in **Figure 1**. The questionnaire included patient demographic characteristics such as age, sex, education, province, and location (urban or rural). Other collected information included income, ethnicity, smoking status (current smoking, current nonsmoking). Height and weight were measured, and BMI (kg/m^2^) was calculated. Waist circumference (WC), systolic blood pressure (SBP), and diastolic blood pressure (DBP) were measured.

### 2.2 Laboratory tests

Fasting blood and urine samples were collected from each participant. The centrifuged serum samples were stored at -20°C. After investigation and collection, all samples were airlifted to the Shenyang Central Laboratory through the cold chain system. Laboratory tests were performed on venous blood samples, which were collected from each subject after an 8-h fast, to measure fasting plasma glucose (FPG), lipid profile, glycosylated hemoglobin (HbA1c). An oral glucose tolerance test (OGTT) was performed in all subjects except those diagnosed with diabetes. We used the hexokinase enzymatic method to measure PG and OGTT–2 h PG (Au400 automatic analyzer, Olympus company, Japan; reagent purchased from Daiichi Pharmaceutical Co. Ltd). Serum triglyceride (TG), total cholesterol (TC), low-density lipoprotein cholesterol (LDL–C), high-density lipoprotein cholesterol (HDL–C) and uric acid (UA) were assessed using reagents from Mindray Company (BS-180). Bio-Rad reagents were used for HbA1c measurement.

### 2.3 Clinical diagnosis

MetS was defined according to the National Cholesterol Education Programme-Adult Treatment Panel III (NCEP-ATP III) (7) as having three or more of the following factors: (1) a waist circumference of ≥90 cm for men and ≥80 cm for women; (2) a systolic blood pressure of ≥130 mmHg or a diastolic blood pressure of ≥85 mmHg or receiving anti-hypertension treatment; (3) a fasting triglyceride level of ≥1.7 mmol/L or receiving corresponding treatment; (4) a high-density lipoprotein cholesterol (HDL-C) level of <1.03 mmol/L for men and <1.30 mmol/L for women or receiving corresponding treatment; and (5) a fasting blood glucose (FBG) level of ≥5.6 mmol/L, receiving anti-diabetes treatment or reporting physician-diagnosed diabetes. Waist circumference, FBG, TG, HDL-C and SBP, as mentioned above, are considered risk components of MetS in this study.

### 2.4 Metabolic syndrome severity score

Patients were assessed with a six-point (0–5) MetS severity score (MSSS) adapted from the IDF and American Heart Association/National Heart, Lung, and Blood Institute (AHA/NHLBI) guidelines (2). A patient’s MSSS was determined by the sum of the following cooccurring components: (1) obesity—body mass index (BMI)> 30 kg/m^2^, (2) DM—current diagnosis or HbA1c ≥ 6.5, (3) diagnosis or treatment of hyperlipidemia (HLD), (4) diagnosis or treatment of hypertension (HTN), and (5) diagnosis or treatment of hypertriglyceridemia (HTG). Each component of the MSSS is weighed equally as is consistent with MetS components when conferring a clinical diagnosis.

### 2.5 Statistical analysis

Estimates were weighted to reflect age, sex, and urban−rural distribution of provinces of the adults living in China. Weighting coefficients were derived from the 2010 Chinese population census data. The weighting coefficient is the inverse of the adjusted probability that the respondent has access to the data. Each case in the analysis is assigned a coefficient (individual weight) that, multiplied by the coefficient, represents the actual population with the same characteristics of sex, age, province, and region. Analysis of disaggregated data expressed as counts and percentages was performed with the use of the chi-square test and Fisher exact test, as appropriate. Continuous data are presented as means ± standard errors (SE), differences between continuous variables were assessed with the use of analysis of variance (ANOVA). The UpSet plot was used to visualize the population distribution of different MetS combinations, and percentage stacked bar plots showed the composition of MSSS scores by gender and age. The restricted cubic spline regression was used to estimate the dose-response relationship between the risk of MSSS or each component and age. This study was analyzed with R (version 4.2.1), mainly invoking the survey, rms and mice packages. In all analyses, P<0.05 was considered statistically significant.

## 3 Results

### 3.1 General characteristics of the TIDE participants

A total of 68383 subjects were ultimately included in this study: 50.35% men and 49.65% women. **Table 1** summarizes the overall characteristics of the subjects included in this study and the corresponding characteristics of different MSSSs. There were significant differences in prevalence among the study groups by sex, income, education level, urban vs. rural residence, ethnicity, smoking status, age group and BMI group. Additionally, there were significant differences in age, BMI, waist circumference, FBG, OGTT-2hPG, HbA1c, TGs, TC, LDL-C, HDL-C, SBP, DBP and UA among the groups (all p<0.0001). Abnormal metabolic indicators such as age, BMI, waist circumference, FBG, OGTT-2hPG, HbA1c, TGs, SBP, and DBP increased with an increasing MSSS. In addition, HDL-C decreased with an increasing MSSS, while TC, LDL-C and UA reached peaks when the MSSS was equal to 4. Subjects with higher MSSS were more likely to have received senior high school education or lower level and were more likely to be Han Chinese relative to ethnic minorities(all p<0.0001). When the MSSS was 0 and 5, the proportion of females was significantly higher than that of males, and the remaining scores were vice versa. In the 18-39 age group, the proportion of the population decreased with an increase in the MSSS, while the trend was opposite in the 60-80 age group; there were relatively more patients with abnormal MetS components in the 40-59 age group than in the other age groups. For those with normal or underweight BMI, the more abnormal MetS components, the smaller the proportion of the population (both men and women), while the opposite is true for those with overweight or obesity (**Supplementary Tables 1, 2**).

**Table 1:** General characteristics of study participants.

Metabolic Syndrome Severity Score								
Altitude (meters)	Total	0	1	2	3	4	5	Pvalue
Age (years)	42.83 (0.10)	34.16 (0.14)	42.15 (0.17)	47.58 (0.22)	50.58 (0.22)	51.91 (0.27)	53.86 (0.66)	< 0.0001
BMI (Kg/m^2^)	24.00 (0.02)	21.34 (0.03)	23.42 (0.03)	25.35 (0.04)	26.79 (0.04)	27.70 (0.06)	28.10 (0.13)	< 0.0001
WC (cm)	83.20 (0.06)	74.35 (0.07)	81.77 (0.09)	87.61 (0.11)	92.12 (0.12)	94.78 (0.15)	95.87 (0.33)	< 0.0001
FBG (mmol/L)	5.42 (0.01)	4.85 (0.00)	5.17 (0.01)	5.57 (0.02)	6.11 (0.02)	6.80 (0.05)	7.56 (0.14)	< 0.0001
OGTT-2h (mmol/L)	6.50 (0.01)	5.50 (0.01)	6.04 (0.02)	6.86 (0.03)	7.93 (0.05)	9.25 (0.09)	10.05 (0.21)	< 0.0001
HbA1c	5.60 (0.01)	5.28 (0.00)	5.44 (0.01)	5.68 (0.01)	6.00 (0.02)	6.38 (0.04)	6.83 (0.09)	< 0.0001
TG (mmol/L)	1.57 (0.01)	0.90 (0.00)	1.27 (0.01)	1.73 (0.01)	2.38 (0.04)	3.27 (0.05)	4.66 (0.16)	< 0.0001
TC (mmol/L)	4.77 (0.01)	4.41 (0.01)	4.69 (0.01)	4.96 (0.01)	5.13 (0.02)	5.35 (0.02)	5.20 (0.06)	< 0.0001
LDL (mmol/L)	2.83 (0.00)	2.51 (0.01)	2.79 (0.01)	3.01 (0.01)	3.12 (0.01)	3.21 (0.02)	2.82 (0.04)	< 0.0001
HDL (mmol/L)	1.46 (0.00)	1.61 (0.00)	1.52 (0.00)	1.41 (0.00)	1.30 (0.01)	1.17 (0.01)	0.88 (0.01)	< 0.0001
SBP (mmHg)	126.28 (0.10)	112.84 (0.11)	123.81 (0.16)	133.33 (0.22)	139.59 (0.27)	143.95 (0.36)	147.88 (0.75)	< 0.0001
DBP (mmHg)	78.39 (0.06)	70.81 (0.08)	77.35 (0.11)	82.27 (0.14)	85.48 (0.17)	88.36 (0.27)	89.74 (0.61)	< 0.0001
UA (mmol/L)	317.95 (0.53)	293.02 (0.93)	310.39 (0.95)	330.34 (1.24)	345.59 (1.30)	359.84 (1.98)	357.60 (4.67)	< 0.0001
Sex (%)								< 0.0001
Male	34431 (50.35)	28714 (41.99)	35190 (51.46)	38342 (56.07)	38082 (55.69)	38130 (55.76)	29097 (42.55)	
Female	33952 (49.65)	39669 (58.01)	33193 (48.54)	30041 (43.93)	30301 (44.31)	30253 (44.24)	39286 (57.45)	
Income (%)								< 0.0001
<30000yuan	30519 (44.63)	27087 (39.61)	29931 (43.77)	32899 (48.11)	33665 (49.23)	33610 (49.15)	34513 (50.47)	
≥30000yuan	37864 (55.37)	41296 (60.39)	38452 (56.23)	35484 (51.89)	34718 (50.77)	34773 (50.85)	33870 (49.53)	
Education (%)								< 0.0001
Senior high school or lower level	45434 (66.44)	34834 (50.94)	44900 (65.66)	51615 (75.48)	54337 (79.46)	55424 (81.05)	57893 (84.66)	
College or higher level	22949 (33.56)	33549 (49.06)	23483 (34.34)	16768 (24.52)	14046 (20.54)	12959 (18.95)	10490 (15.34)	
Location (%)								< 0.0001
Urban	35258 (51.56)	39669 (58.01)	34622 (50.63)	32461 (47.47)	32728 (47.86)	32796 (47.96)	31183 (45.60)	
Rural	33125 (48.44)	28714 (41.99)	33761 (49.37)	35922 (52.53)	35655 (52.14)	35587 (52.04)	37200 (54.40)	
Ethnic (%)								< 0.0001
Ethnic han	65402 (95.64)	64560 (94.41)	65265 (95.44)	65709 (96.09)	66208 (96.82)	66824 (97.72)	67761 (99.09)	
Others	2981 (4.36)	3823 (5.59)	3118 (4.56)	2674 (3.91)	2175 (3.18)	1559 (2.28)	622 (0.91)	
Smoke (%)								< 0.0001
NO	52922 (77.39)	57066 (83.45)	53277 (77.91)	50638 (74.05)	49072 (71.76)	48094 (70.33)	51274 (74.98)	
YES	15461 (22.61)	11317 (16.55)	15106 (22.09)	17745 (25.95)	19311 (28.24)	20289 (29.67)	17109 (25.02)	
Age (%)								< 0.0001
20-39	31128 (45.52)	47957 (70.13)	32106 (46.95)	22047 (32.24)	16357 (23.92)	13314 (19.47)	11700 (17.11)	
40-59	25733 (37.63)	16870 (24.67)	26184 (38.29)	30164 (44.11)	32701 (47.82)	34684 (50.72)	32208 (47.10)	
60-80	11523 (16.85)	3556 (5.20)	10093 (14.76)	16173 (23.65)	19325 (28.26)	20385 (29.81)	24474 (35.79)	
BMI (%)								< 0.0001
<18	3488 (5.10)	8281 (12.11)	2681 (3.92)	992 (1.45)	321 (0.47)	109 (0.16)	89 (0.13)	
18-25	39532 (57.81)	54652 (79.92)	46576 (68.11)	31538 (46.12)	19646 (28.73)	13177 (19.27)	10497 (15.35)	
25-30	21103 (30.86)	5334 (7.80)	17397 (25.44)	30150 (44.09)	37939 (55.48)	40845 (59.73)	41433 (60.59)	
>30	4260 (6.23)	123 (0.18)	1730 (2.53)	5710 (8.35)	10476 (15.32)	14244 (20.83)	16364 (23.93)	

Data are presented as mean ± standard error (SE) or percentage. ¥30000=£3365; €3849; $4115.

BMI, body mass index; WC, waist circumference; FBG, fasting blood glucose; OGTT, oral glucose tolerance test; HbA1c, glycosylated hemoglobin; TG, triglyceride; TC, total cholesterol; LDL-C, low density lipoprotein cholesterol; HDL-C, high density lipoprotein cholesterol; SBP, systolic blood pressure; DBP, diastolic blood pressure; UA, uric acid.

### 3.2 Characteristics of MetS components and their clustering in the population

**Figure 2:**
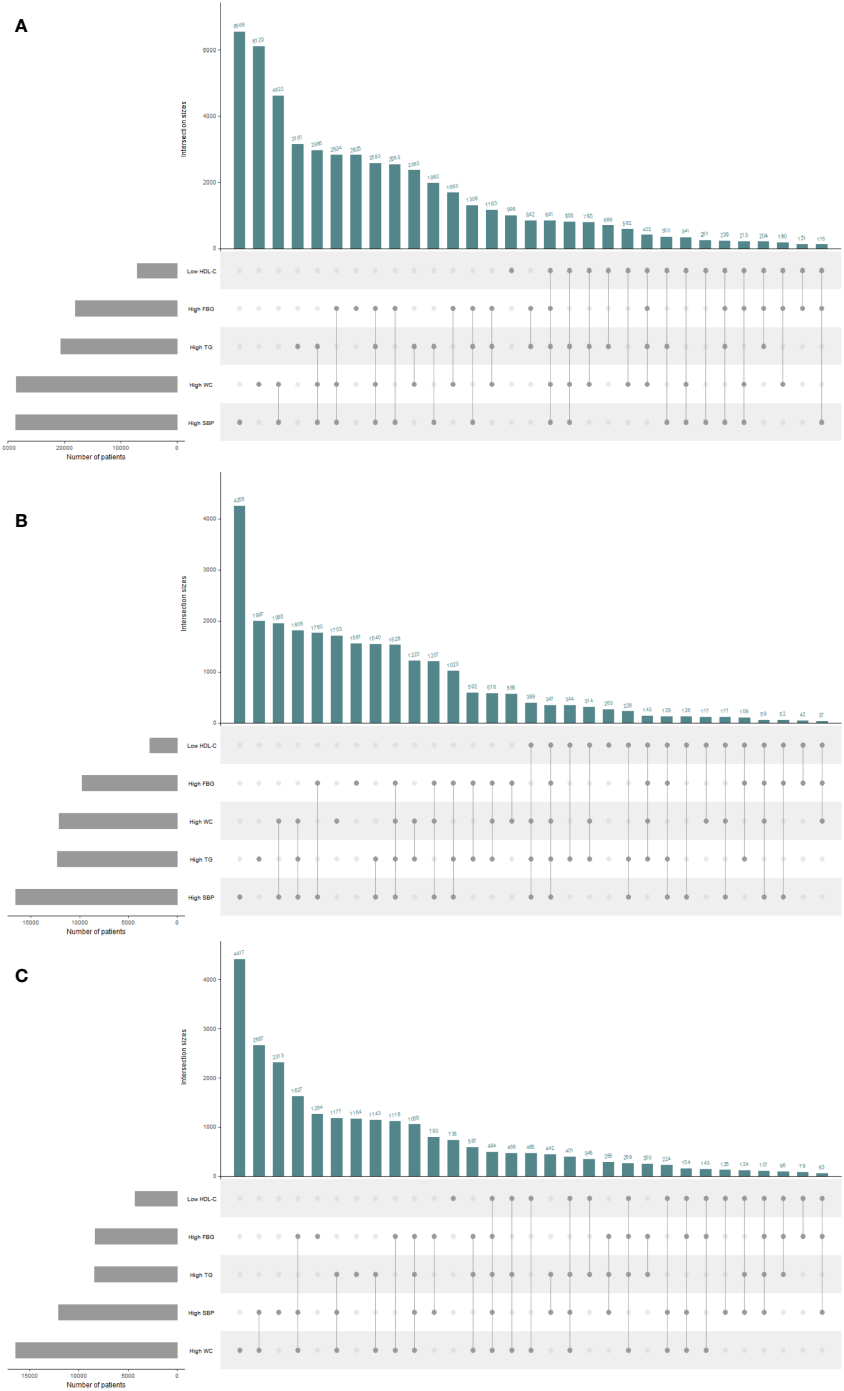
UpSet plots for all metabolic syndrome (MetS) components clustering condition in the total population and different genders. The gray strip at the bottom left shows the number of subjects included in each MetS component. Dark gray dots at the bottom right indicate the MetS abnormalities corresponding to the line, dots and lines indicate one combination of abnormal MetS component aggregation. Histograms represent the number of patients in each subset. **(A)** The total population. **(B)** The male population. **(C)** The female population. HDL-C, high density lipoprotein cholesterol; WC, waist circumference; FBG, fasting blood glucose; TG, triglyceride; SBP, systolic blood pressure.

The distributions of the risk components of MetS in the total population and in men and women are shown in **Figure 2**; **Table 2**. Moreover, as shown in **Table 2**, there were significant differences in the weighted prevalence under the aggregation of different components of MetS for the total population, males, and females. Among the 68383 participants, 17688 had no abnormal components; 50695 (26113 men and 24582 women) had abnormal components of MetS, the breakdown of which was as follows: MSSS 1 = 19672 (26.99%), MSSS 2 = 15688 (21.53%), MSSS 3 = 10260 (14.19%), MSSS 4 = 4234 (5.96%), and MSSS 5 = 841 (1.2%). In the total population (**Figure 2A**), The top three combinations with the highest incidence under the aggregation of metabolic abnormal components were high SBP (9.41%, n=6568), high WC (8.13%, n=6120), and the cooccurrence of high SBP and high WC (6.33%, n=4622). In men (**Figure 2B**), the prevalence of3high SBP was the highest in all groups (12.3%, n=4255). Besides high SBP, the remaining top three combinations with relatively high prevalence were high TG (5.46%, n=1997), the combination of high WC and high SBP (5.71%, n=1955), and the combination of high TG, high WC and high SBP (4.93%, n=1808). The proportion of women with elevated WC was the highest (11.54%, n=4417), which significantly differed from other groups (**Figure 2C**). The remaining higher-prevalence groups were the combination of high SBP and high WC (6.95%, n=2667) and the group with elevated SBP only (6.48%, n=2313). However, in all various combinations of risk components, the prevalence of combinations containing decreased HDL-C was relatively low, ranging from 0.11% to 1.2% in the total population, and similar in men and women.

**Table 2:** The distribution of metabolic syndrome risk components.

Variable	Total (%)	Man (%)	Woman (%)	Pvalue	MSSS
00000	50695 (30.15)	26113 (25.15)	24582 (35.22)	< 0.0001	0
00001	6120 (8.13)	1703 (4.76)	4417 (11.54)	< 0.0001	1
00010	998 (1.06)	263 (0.51)	735 (1.63)	< 0.0001	1
00011	582 (0.74)	117 (0.35)	465 (1.14)	< 0.0001	2
00100	3161 (4.27)	1997 (5.46)	1164 (3.06)	< 0.0001	1
00101	2363 (3.06)	1220 (3.34)	1143 (2.78)	< 0.0001	2
00110	689 (0.91)	344 (0.97)	345 (0.84)	< 0.0001	2
00111	780 (1.07)	314 (0.90)	466 (1.23)	< 0.0001	3
01000	2825 (4.12)	1561 (4.56)	1264 (3.68)	< 0.0001	1
01001	1683 (2.28)	565 (1.68)	1118 (2.89)	< 0.0001	2
01010	121 (0.15)	42 (0.10)	79 (0.21)	< 0.0001	2
01011	180 (0.27)	37 (0.10)	143 (0.44)	< 0.0001	3
01100	842 (1.19)	592 (1.68)	250 (0.69)	< 0.0001	2
01101	1163 (1.56)	576 (1.64)	587 (1.48)	< 0.0001	3
01110	204 (0.30)	108 (0.30)	96 (0.30)	< 0.0001	3
01111	402 (0.58)	143 (0.43)	259 (0.72)	< 0.0001	4
10000	6568 (9.41)	4255 (12.30)	2313 (6.48)	< 0.0001	1
10001	4622 (6.33)	1955 (5.71)	2667 (6.95)	< 0.0001	2
10010	251 (0.27)	126 (0.27)	125 (0.26)	< 0.0001	2
10011	341 (0.46)	117 (0.29)	224 (0.62)	< 0.0001	3
10100	1982 (2.86)	1540 (4.55)	442 (1.15)	< 0.0001	2
10101	2985 (3.93)	1808 (4.93)	1177 (2.92)	< 0.0001	3
10110	350 (0.48)	226 (0.71)	124 (0.24)	< 0.0001	3
10111	800 (1.08)	399 (1.12)	401 (1.03)	< 0.0001	4
11000	2553 (3.74)	1760 (5.33)	793 (2.14)	< 0.0001	2
11001	2834 (4.10)	1207 (3.66)	1627 (4.56)	< 0.0001	3
11010	115 (0.11)	52 (0.10)	63 (0.12)	< 0.0001	3
11011	213 (0.29)	59 (0.15)	154 (0.42)	< 0.0001	4
11100	1308 (1.91)	1023 (3.05)	285 (0.75)	< 0.0001	3
11101	2583 (3.71)	1528 (4.52)	1055 (2.87)	< 0.0001	4
11110	236 (0.30)	129 (0.35)	107 (0.24)	< 0.0001	4
11111	841 (1.20)	347 (1.02)	494 (1.39)	< 0.0001	5

The numerical arrangement of the variable column represents the arrangement of metabolic syndrome components, from the first to the fifth order being systolic blood pressure, fasting blood glucose, triglyceride, high density lipoprotein cholesterol, waist circumference. The number 1 indicates that the component is not within the normal reference range, and 0 indicates that the component is within the normal reference range.

MSSS, metabolic syndrome severity score.

### 3.3 Rate ratio of the five components at different MSSSs

**Figure 3:**
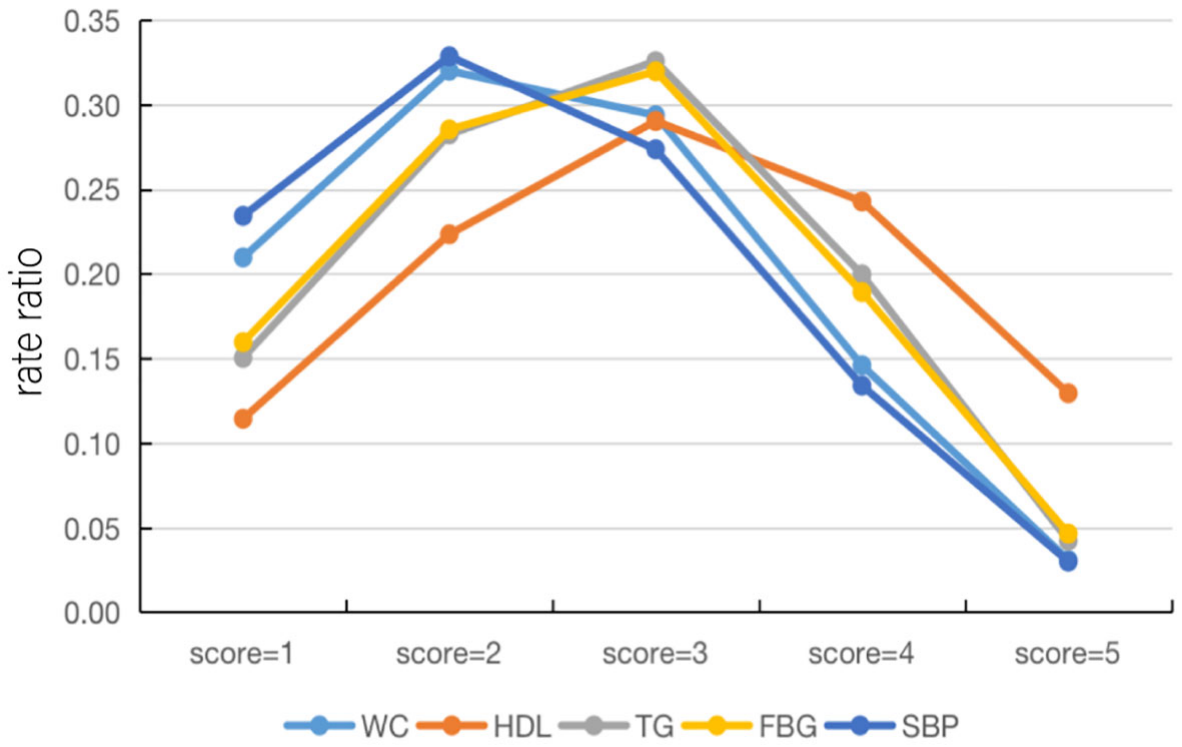
Line plots of the rate ratios of each component under the MSSS grouping. HDL-C, high density lipoprotein cholesterol; WC, waist circumference; FBG, fasting blood glucose; TG, triglyceride; SBP, systolic blood pressure; MSSS, metabolic syndrome severity score.

The total prevalence of increased WC was 38.79% in all combinations of risk components and 8.13% in MSSS 1. Thus, the proportion of increased WC under the classification of MSSS 1 in all combinations was 20.96%, which indicated the rate ratio of increased WC under this classification and facilitated a clearer comparison with the remaining components. **Supplementary Table 3** showed how this method was used to analyze all five components in different aggregation cases, and **Figure 3** showed line plots to make the data changes more visible. When the MSSS=1 or 2, the rate ratios of elevated SBP and high WC were highest, the rate ratio of decreased HDL-C was lowest. When the MSSS=3, the rate ratios for elevated TG and elevated FBG became the highest, although this number varied only slightly for the five components. However, when the MSSS=4 or 5, the rate ratio of low HDL-C became the highest, and the rate ratios of high TG and high FBG were higher than those of high WC and high SBP. In this way, low HDL-C was considered to exist when more components are aggregated.

### 3.4 Characteristics of MetS components and the MSSS by age stratification

**Figure 4:**
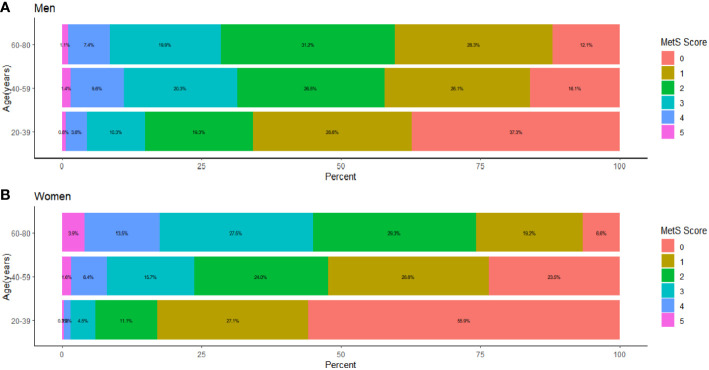
The proportion distribution of age stratified and sex classified population under each MSSS group, The population was stratified by age as 18-39 years old, 40-59 years old, and 60-80 years old. **(A)** The proportion distribution of male population in three age groups under each MSSS group. **(B)** The proportion distribution of female population in three age groups under each MSSS group.

**Figure 5:**
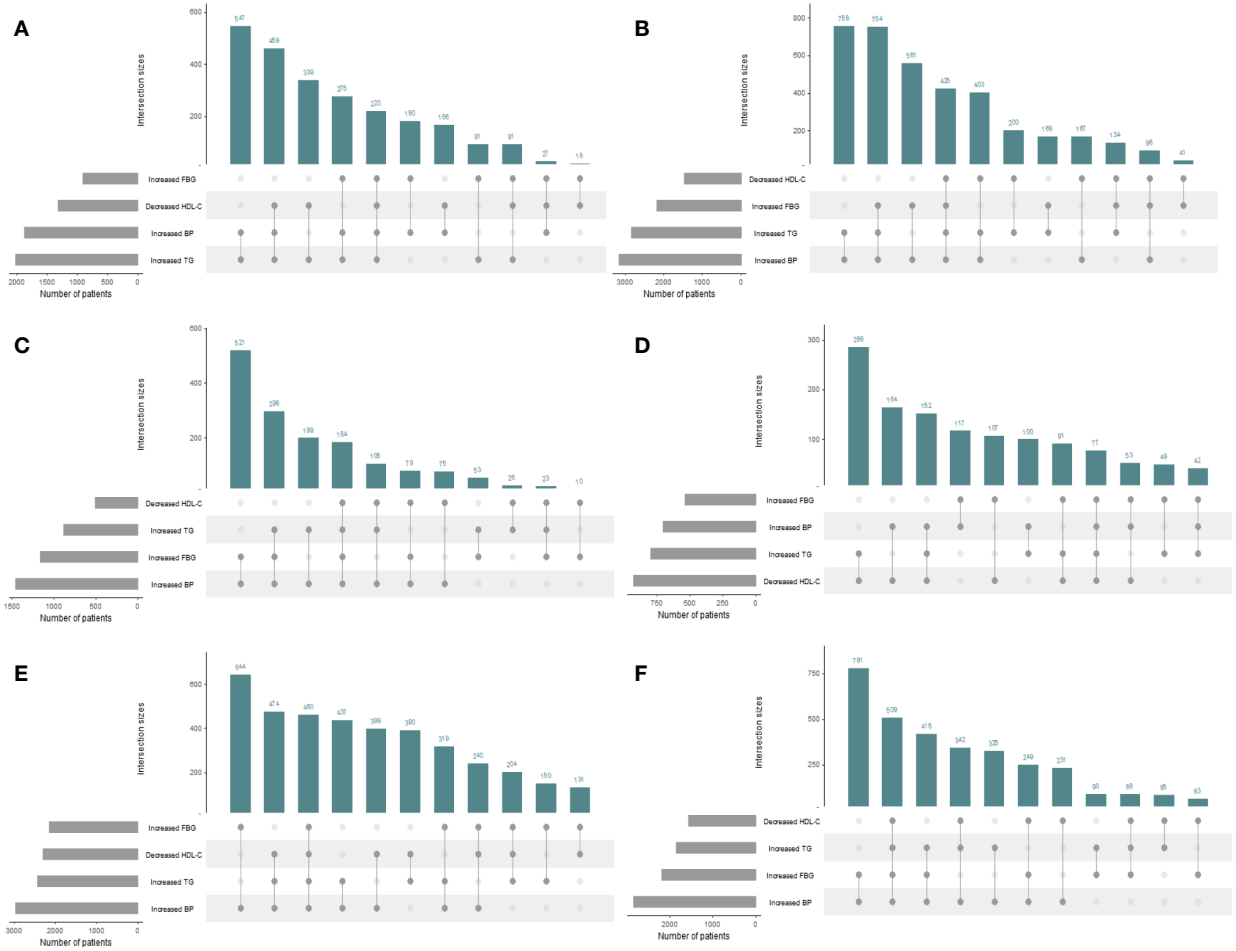
UpSet plots for metabolic syndrome (MetS) components clustering condition except waist circumference in the total population and different genders and 3 age groups. The gray strip at the bottom left shows the number of subjects included in each MetS component. Dark gray dots at the bottom right indicate the MetS abnormalities corresponding to the line, dots and lines indicate one combination of abnormal MetS component aggregation. Histograms represent the number of patients in each subset. Waist circumference was excluded under the IDF definition of “obesity-centered” metabolic syndrome. **(A)** Men aged 18-39 years; **(B)** Men aged 40-59 years; **(C)** Men aged 60-80 years; **(D)** Women aged 18-39 years; **(E)** Women aged 40-59 years; **(F)** Women aged 60-80 years.

**Figure 4** presents the proportion of people with different MSSSs in three age groups (20-39, 40-59, 60-80 years) for men and women by percentage stacked bars charts. In the 20-39 age group, the prevalence in men was significantly higher than that in women when 1 to 5 risk components were gathered; that is, the proportion of women without abnormal metabolic components was significantly higher than that of men. In the 40-59 age group, the prevalence of males was significantly higher than that of females when the MSSS=2,3,4, and the opposite was observed in other groups. In the 60-80 age group, when the MSSS was ≥ 3, the prevalence of women was significantly higher than that of men, while the opposite was true when the MSSS=1 or 2. We could see that when women entered middle and old age, when more than three metabolic risk factors were clustered, the prevalence increased significantly and was significantly higher than that of men. Regarding each component, as shown in **Figure 5**, according to the IDF’s “obesity-centered” definition of MetS, after excluding high WC, in men of 18-59 age group, the combination of elevated TG and SBP was the most prevalent, indicating that men were most affected by these two risk components (**Figures 5A, B**). Elevated FBG and SBP turned to the most influencing factors in men of 60-80 age group (**Figure 5C**). Women aged 20-39 years had the highest number of patients with a combination of high TG and low HDL-C (**Figure 5D**), which was significantly higher than those in the other age groups. In the 40-59 and 60-80 female age groups, the combination of high SBP and high FBG was the most common (**Figures 5E, F**).

### 3.5 The nonlinear relation between age and risk of disease shown by restricted cubic splines

**Figure 6:**
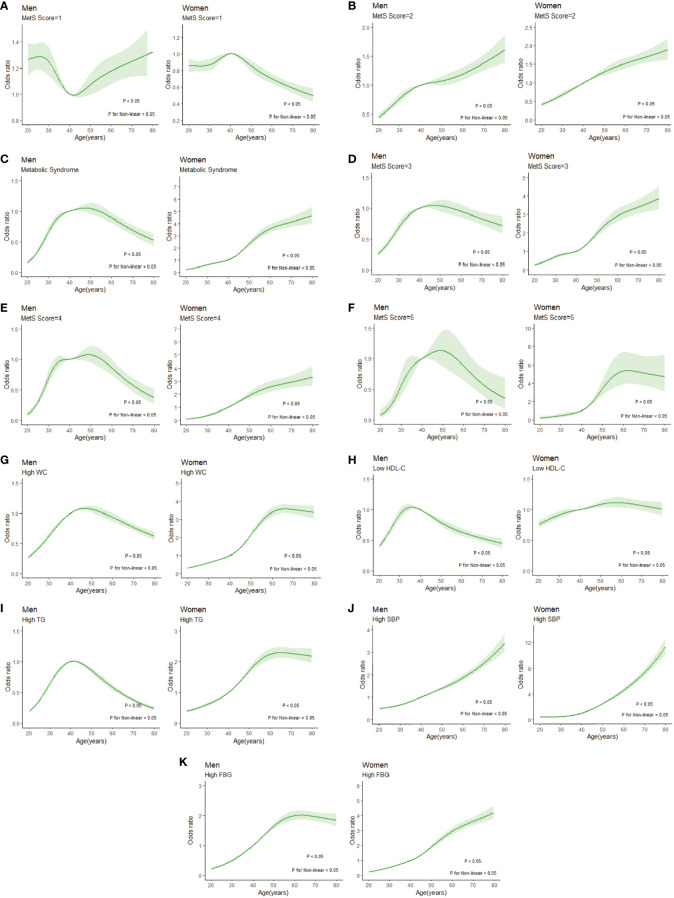
Restricted cubic splines of nonlinear relationships between age and risk of disease under different MSSSs or metabolic syndrome components. **(A)** MSSS=1; **(B)** MSSS=2; **(C)** metabolic syndrome; **(D)** MSSS=3; **(E)** MSSS=4; **(F)** MSSS=5; **(G) **high WC; **(H) **low HDL-C; **(I) **high TG; **(J)** high SBP; **(K)** high FBG. WC, waist circumference; HDL-C, high density lipoprotein cholesterol; TG, triglyceride; SBP, systolic blood pressure; FBG, fasting blood glucose; MSSS, metabolic syndrome severity score.

In **Figure 6**, we used restricted cubic splines to flexibly model and visualize the relation of age with the prevalence of different MSSSs or the specific components. When the MSSS=1(**Figure 6A**), the prevalence of males decreased from 30 to 40 years old and then increased. In contrast, the prevalence of females peaked at 40 to 50 years old, began to decline rapidly and then gradually levelled off. When the MSSS=2 (**Figure 6B**), the overall prevalence showed a similar upward trend both in men and women. Among the population with MetS (**Figures 6C-F**), it showed an inverted U-shaped curve with age and peaked at age 40-60 years in men, while the prevalence of females consistently increased with age, and it increased rapidly from the age of 40-60 years (P for nonlinearity <0.05). Regarding each specific component, for women of all age groups (**Figures 6G–K**), the prevalence of each abnormal component basically increased with age. In male population, the prevalence of high TG, high WC and low HDL-C showed an inverted U-shaped curve with age (**Figures 6G-I**). For high SBP and high FBG (**Figures 6J, K**), the prevalence of the male population increased with age.

## 4 Discussion

Based on data from the TIDE study, this analysis provides up-to-date information on the epidemiological characteristics of risk factors for MetS in Chinese adults. Understanding the prevalence of each abnormal component and its clustering will help identify individuals at higher risk for metabolic abnormalities. Among 68383 participants, 50695 (26113 men and 24582 women) had abnormal components of MetS. There were significant differences in the demographic characteristics between the total population and the 6 MSSS groups. Subjects with more abnormal MetS components were less educated and more likely to be of Han ethnicity (p<0.0001). The top three clustering of abnormal metabolic components were high SBP (9.41%, n=6568), high WC (8.13%, n=6120), and the cooccurrence of high SBP and high WC (6.33%, n=4622). The highest incidence factors were high SBP in the male population and high WC in the female population. These two variables and their coexistence are important components of MetS and should be addressed as key factors for MetS prevention and treatment. We also found that in the presence of low HDL-C, having all five MetS components was more likely to occur. Restricted cubic splines showed that when the MSSS was ≥3, the prevalence of the male population peaked at 40-60 years old, while the prevalence of the female population increased most rapidly in this age group, therefore, 40-60 age group can be considered as a high-risk period of MetS. The peak age of having low HDL-C was earlier (30-40 years old) in men than in women. The prevalence of high SBP and high FBG increased with age in males. The prevalence of each abnormal component increased with age in women of all age groups.

The clustering characteristics of components of MetS in Chinese adults were related to socioeconomic status and lifestyle. Sex, income, education level, ethnicity (Han or other), smoking status, age, and BMI were associated with MetS prevalence. There was a higher MetS prevalence in men than in women when the MSSS was 1-4, but the opposite was true when the MSSS was equal to 5. The sex distribution was also found to be significantly different between the groups (MSSS 0 = 51.7% male, MSSS 1 = 46.9% male, MSSS 2 = 62.5% male, MSSS 3 = 57.9% male, MSSS 4 = 72.2% male, and MSSS 5 = 52.6% male; p = 0.039) in another study (20). The group in the low-income tier represented a greater proportion than the group with high income when the MSSS≥2, unlike men, which was similar to the findings of Kim H (21) and Drewnowski A (22). Azra Bureković et al. (23) conducted a study of participants aged 18 to 70 years in Sarajevo, showing that those with MetS had lower levels of education and less motivation to make lifestyle changes. Jeong Suk Jeon et al. (24) found that a two-day multidisciplinary educational camp improved MetS indicators and improved mood status in patients with MetS. A study (25) of middle-aged and elderly people with MetS in urban Portugal showed that the risk of MetS increased with age and decreased with education level in women. Similarly, in this study, the higher the MSSS, the greater the proportion of the population with lower education, and this finding was observed in both men and women. Smoking was significantly associated with higher MetS risk, which is consistent with previous studies (26), and this could be observed in both conventional and e-cigarette users (27). In this study, the proportion of the Han population increased with increasing MSSS. The prevalence of MetS differs among certain predefined racial/ethnic groups (28); for instance, the prevalence of MetS is highest in Hispanics and lowest in African Americans in the United States (29). An earlier study reported MetS to be highest in people of indigenous ancestry, followed by South Asians, Europeans, and East Asians (30). The prevalence of MetS in the 6 MSSS groups tended to increase with increasing age, which was similar to the findings of Yao F et al. (11). BMI is the most feasible and widely used criterion to identify and classify patients with overweight or obesity and is also a significant independent predictor of the presence of MetS (31). This study found that BMI increased with MSSS, and rates of overweight and obesity also increased with the MSSS. Kobo O et al. (32) found that BMI as a single survey measurement of obesity offers high negative predictive value for MetS. A recent study (33) in young Chinese adults suggested a lower upper limit of normal BMI of 24.4 kg/m^2^ in men and 23.8 kg/m^2^ in women, or the use of a (waist-to-hip) height ratio to screen for obesity more accurately, but we failed to rescreen for obesity with this or a similar approach because the diagnostic criteria for MetS have not changed. This study did not identify any correlations between diet or lifestyle and the risk of MetS. This lack of correlation may have been related to bias.

This study shows that high TG and high FBG are more likely to occur when multiple MetS components are aggregated, and when one’s HDL-C is low, it is more likely that they will have all five MetS components. This suggests that the presence of low HDL-C may be associated with many adverse health conditions. Elevated TGs and reduced HDL-C are associated with pelvic organ prolapse severity in postmenopausal women, as Gava G et al. (34) concluded. One study indicated that MetS and some of its components (low HDL-C and the number of MetS components) seem to be risk factors for stroke recurrence (12). In another study (17), HDL-C was found to be associated with an increased risk of death related to endometrial cancer (HR = 2.2, P = 0.034) in the multivariate-adjusted model. Feinkohl I et al. (35) found low HDL-C as an independent risk marker of cognitive impairment in older age, whereas BMI, TG, glucose and HbA1c were not. HDL-C may be involved in the accelerated ageing process regarding metabolic disturbance in major depressive disorder only from Huang’s study (36). Chen’s study (37) concluded that low HDL-C and elevated FBG were associated with all-cause and cardiovascular mortality. Similarly, Yen YF et al. (38) found that hypertension and low HDL-C were predictors of cardiovascular disease (CVD) mortality and expanded CVD mortality and, compared with Mets, were associated with a higher risk of expanded CVD mortality. Our findings together with those of the above studies suggest that low HDL-C, especially among elderly individuals, is an important predictor of disease risk, not just MetS, and is associated with adverse outcomes to some extent, as well as high TGs and high FBG.

Fernández-Verdejo R et al. (39) proposed that the order in which abnormal components accumulate may help to identify the underlying pathophysiological mechanisms and improve the treatment of MetS. A hierarchical order for organ failure has been proposed depending on the organ’s susceptibility. Thus, different susceptibilities of the liver (regulating HDL-C and triglycerides), endothelium (regulating blood pressure), and pancreas (regulating glycemia) could determine the accumulation of MetS components. They found that the most frequent sequence of accumulation of MetS components was [i] elevated waist circumference, [ii] reduced HDL-C, [iii] elevated triglycerides, [iv] elevated blood pressure, and [v] elevated glucose. In approximately half of the subjects, MetS appears to develop following an accumulation of abdominal obesity that subsequently leads to liver dysfunction. Additionally, the organism seems to preserve glycemia within the normal range as long as possible. Franco OH et al. (40) grouped MetS components into pairs and determined the order of appearance, and HDL-C preceded the appearance of any other component in the overall sample. However, it may have altered the natural history of MetS for including subjects who were medically treated for elevated blood pressure. According to the method proposed by Fernandez-Verdejo R, this study showed that the cumulative order of MetS components in the Chinese population was [i] elevated SBP, [ii] elevated WC, [iii] elevated TGs, [iv] elevated FBG, and [v] reduced HDL-C. There are slight discrepancies with previous studies because MetS is a heterogeneous and complex syndrome. The presence of insulin resistance, obesity, activation of the sympathetic nervous system and sodium retention are the prevailing mechanisms of hypertension (41). Obesity-related hypertension may be the result of a combination or overlap of several of these factors. Previous research has also demonstrated that WC was positively associated with the prevalence of hypertension (42). Other studies have concluded that WC cut-off points represent values for epidemiological identification of risk for hypertension. This suggests that further understanding of the development and progression of MetS and its components is needed. Certain trajectories and combinations of components may confer higher risks of incident CVD and mortality. Future efforts should focus on preventing and managing individual risk factors rather than on diagnosing Mets in elderly individuals.

The restricted cubic splines showed that the prevalence of MetS in women increased with age, especially after the age of 40 years, which was different from the inverted U-shaped curve in men (peak at 40-60 years). The prevalence of a MSSS≥3 was significantly higher in women than in men aged 60-80 years. The same trend was found in Kim H’s study from the 2012 National Health Screening Data in Korea.

Overall, the prevalence of MetS is higher in women than in men. In many countries studied, including China, this sex difference emerges in midlife (Foucan L et al. (43); Park E (44); Li W (45)). Franco et al. (40) found that, similar to our findings, central obesity is a factor that seems to affect women more than men. Hypertension is also the most common factor in the development of MetS, and in men, hypertension plays a dominant role. This may reflect a faster rate of accumulation of visceral fat in middle-aged women, which appears to begin at a later age compared with men. Menopause nearly adversely affects all components of MetS (46). This is caused by a change in hormonal secretion due to a decrease in ovarian function. From the menopausal transition to menopause, diminished ovarian function leads to hormonal changes characterized by decreases in reproductive hormones, including estrogen, progesterone, testosterone, and inhibin B (47). Depletion of ovarian reserve during the menopausal transition raises follicle-stimulating hormone (FSH) markedly. Estrogens regulate various aspects of glucose and lipid metabolism and adipokine secretion, and estrogen deficiency leads to increased obesity and abdominal obesity, hyperlipidemia, impairment of glucose metabolism, and insulin resistance (48). In some studies, FSH levels were inversely correlated with the presence of MetS (49, 50). More than one-third of women’s lives are spent in menopause. As life expectancy gradually increases, life expectancy after menopause has further increased. In addition, health and disease status are expected to worsen with age, and health care for middle-aged women with hypermetabolic syndrome will become even more important. Raczkiewicz D et al. (51) found that the prevalence of MetS and some of its criteria depended on BMI, body fat accumulation, parity, severity of menopausal symptoms and lack of physical activity. This suggests that more severe metabolic disorders in middle-aged and older women may also result from decreased physical activity, a high-calorie diet, and psychological changes such as anxiety and depression after menopause. Women with obesity showed lower self-esteem and higher depression tendencies than normal-weight individuals. Psychological changes, in turn, can exacerbate unhealthy eating habits. Asian women usually carry greater abdominal and visceral fat than Caucasian women with a similar body mass index (BMI) (52). Middle-aged and older female residents who engage in daily physical activity in a semimountainous area of Japan may experience protective effects against MetS compared with those who do not exercise (53). In addition, the development of insulin resistance in older populations may play a role (54). Among study individuals who were middle-aged Korean women, MetS was highly related to the risk of breast cancer (55). To address the risk factors mentioned above and reduce the occurrence of worse health status, it is necessary to control weight, increase physical activity, and go on a healthy diet. At the same time, it is also important to carry out education initiatives and interventions to prevent MetS before menopause. A study conducted by Liu S et al. (56) indicated that the intake of calcium and dairy products may be associated with a lower prevalence of MetS in middle-aged and older women.

The survey data were nationally representative, were obtained from the TIDE project database, covered all provinces in mainland China and had a sufficient sample size and representativeness. After weighted adjustment, this study better reflected the epidemiological characteristics of MetS components in Chinese residents aged ≥18 years. Under the common definition of MetS, it is not a unique entity but a series of MetS components that may have selective risks for adverse outcomes such as cardiovascular risk and cancer. This study analyzed the characteristics of the components and their clustering of MetS in the Chinese population to provide some evidence for disease risk control and prevention in the future. In addition, the epidemiological characteristics of each component were further analyzed by age stratification and sex classification. Our study also has some limitations. First, this is a cross-sectional study, causality cannot be established, and the cumulative order of disease as proposed in the previous study needs further prospective investigation. Second, we could not evaluate the effect of the different MetS components completely independent of other risk factors for CVD and mortality, such as alcohol consumption and socioeconomic status, because data for these factors were incomplete. Another limitation is that TIDE participants were Chinese; therefore, our results may not apply to other ethnic groups, and any generalization should be considered with caution.

## 5 Conclusions

In our study, more than 70% of the Chinese adults have abnormal MetS components. The 40-60 years old age group can be regarded as the high-risk period of MetS, and the risk of women increased significantly with age. The top three components’ combinations with the most incidence were high SBP, high WC, and their combination. When HDL-C decreases, the aggregation of multiple components is more likely to occur. Therefore, prevention and treatment of MetS requires early screening and education, as well as focusing on its components.

## Data availability statement

The original contributions presented in the study are included in the article/**Supplementary Material**. Further inquiries can be directed to the corresponding author.

## Ethics statement

The studies involving humans were approved by Public Welfare, National Health and Family Planning Commission of China (Grant No. 201402005). The studies were conducted in accordance with the local legislation and institutional requirements. The participants provided their written informed consent to participate in this study.

## Author contributions

XZ: Formal analysis, Investigation, Writing – original draft, Writing – review & editing. CL: Data curation, Methodology, Visualization, Writing – review & editing. BS: Formal analysis, Investigation, Writing – review & editing. DC: Data curation, Investigation, Writing – review & editing. DT: Project administration, Supervision, Writing – review & editing. ZS: Supervision, Writing – review & editing. WT: Supervision, Writing – review & editing.
