# Supplementary material for: The prevalence and clustering of metabolic syndrome risk components in Chinese population: a cross-sectional study
Source: Front Endocrinol (Lausanne). 2023 Dec 13;14:1290855. doi: 10.3389/fendo.2023.1290855 (PMC10751355; doi:10.3389/fendo.2023.1290855)
Supplement: Supplementary file 1 [file Table_1.docx]

Supplementary Material

**The prevalence and clustering of Metabolic Syndrome risk components in Chinese population: a cross-sectional study**

**Xu Zhao**^1†^**, Cihang Lu**^1†^**, Bo Song**^1^**, Deshi Chen**^1^**, Di Teng**^1*^**, Zhongyan Shan**^1^**, Weiping Teng**^1^

*** Correspondence:** Di Teng, 18698818800@163.com

# Supplementary Tables

**Supplementary Table 1. General characteristics of women**

|  |  | | | **Metabolic Syndrome Severity Score** | | | |  | | |
| --- | --- | --- | --- | --- | --- | --- | --- | --- | --- | --- |
| **Altitude（meters）** | **Total** | **0** | **1** | | **2** | **3** | **4** | | **5** | **Pvalue** |
| Age(years) | 43.03(0.13) | 33.61(0.17) | 41.85(0.25) | | 49.64(0.24) | 54.24(0.34) | 56.93(0.41) | | 58.75(0.73) | < 0.0001 |
| BMI(Kg/㎡) | 23.41(0.03) | 20.97(0.03) | 23.20(0.05) | | 25.02(0.06) | 26.34(0.07) | 26.77(0.09) | | 27.49(0.17) | < 0.0001 |
| WC(cm) | 79.76(0.08) | 71.55(0.08) | 79.55(0.13) | | 85.02(0.14) | 89.05(0.16) | 90.60(0.20) | | 93.39(0.44) | < 0.0001 |
| FBG(mmol/L) | 5.33(0.01) | 4.84(0.01) | 5.10(0.01) | | 5.46(0.02) | 6.11(0.04) | 6.78(0.08) | | 7.53(0.18) | < 0.0001 |
| OGTT-2h(mmol/L) | 6.50(0.02) | 5.56(0.02) | 6.10(0.02) | | 6.99(0.04) | 8.10(0.07) | 9.52(0.14) | | 10.32(0.26) | < 0.0001 |
| HbA1c | 5.55(0.01) | 5.26(0.01) | 5.40(0.01) | | 5.64(0.02) | 6.01(0.02) | 6.42(0.05) | | 6.93(0.12) | < 0.0001 |
| TG(mmol/L) | 1.36(0.01) | 0.85(0.00) | 1.17(0.01) | | 1.53(0.02) | 1.99(0.02) | 2.85(0.05) | | 4.01(0.18) | < 0.0001 |
| TC(mmol/L) | 4.75(0.01) | 4.44(0.01) | 4.68(0.02) | | 4.98(0.02) | 5.11(0.02) | 5.34(0.04) | | 5.16(0.06) | < 0.0001 |
| LDL(mmol/L) | 2.76(0.01) | 2.46(0.01) | 2.73(0.01) | | 2.99(0.01) | 3.10(0.02) | 3.20(0.03) | | 2.87(0.05) | < 0.0001 |
| HDL(mmol/L) | 1.56(0.00) | 1.69(0.00) | 1.60(0.01) | | 1.51(0.01) | 1.40(0.01) | 1.23(0.01) | | 0.95(0.01) | < 0.0001 |
| SBP(mmHg) | 122.67(0.14) | 110.30(0.13) | 119.62(0.21) | | 131.05(0.31) | 139.04(0.44) | 144.07(0.57) | | 148.35(1.04) | < 0.0001 |
| DBP(mmHg) | 76.04(0.08) | 70.12(0.10) | 75.34(0.15) | | 80.25(0.19) | 82.63(0.24) | 85.17(0.41) | | 87.16(0.67) | < 0.0001 |
| UA(mmol/L) | 272.84(0.59) | 255.63(0.89) | 266.57(1.26) | | 281.68(1.25) | 297.85(1.70) | 313.11(2.62) | | 325.27(5.50) | < 0.0001 |
| Income(%) |  |  |  | |  |  |  | |  | < 0.0001 |
| <30000yuan | 32981(48.23) | 27004(39.49) | 32249(47.16) | | 37009(54.12) | 40975(59.92) | 40510(59.24) | | 41666(60.93) |  |
| ≥30000yuan | 35402(51.77) | 41379(60.51) | 36134(52.84) | | 31374(45.88) | 27408(40.08) | 27873(40.76) | | 26717(39.07) |  |
| Education (%) |  |  |  | |  |  |  | |  | < 0.0001 |
| Senior high school or lower level | 46145(67.48) | 32988(48.24) | 46528(68.04) | | 55165(80.67) | 60280(88.15) | 61285(89.62) | | 62181(90.93) |  |
| College or higher level | 22238(32.52) | 35395(51.76) | 21855(31.96) | | 13218(19.33) | 8103(11.85) | 7098(10.38) | | 6202(9.07) |  |
| Location (%) |  |  |  | |  |  |  | |  | < 0.0001 |
| Urban | 35053(51.26) | 40825(59.70) | 34937(51.09) | | 30554(44.68) | 28680(41.94) | 30403(44.46) | | 28502(41.68) |  |
| Rural | 33330(48.74) | 27558(40.30) | 33446(48.91) | | 37829(55.32) | 39703(58.06) | 37980(55.54) | | 39881(58.32) |  |
| Ethnic(%) |  |  |  | |  |  |  | |  | < 0.0001 |
| Ethnic han | 65484(95.76) | 64725(94.65) | 65402(95.64) | | 65832(96.27) | 66366(97.05) | 67009(97.99) | | 67720(99.03) |  |
| Others | 2899(4.24) | 3658(5.35) | 2981(4.36) | | 2551(3.73) | 2017(2.95) | 1374(2.01) | | 663(0.97) |  |
| Smoke(%) |  |  |  | |  |  |  | |  | < 0.0001 |
| NO | 67303(98.42) | 67562(98.80) | 67432(98.61) | | 67070(98.08) | 67077(98.09) | 66393(97.09) | | 66858(97.77) |  |
| YES | 1080(1.58) | 821(1.20) | 951(1.39) | | 1313(1.92) | 1306(1.91) | 1990(2.91) | | 1525(2.23) |  |
| Age(%) |  |  |  | |  |  |  | |  | < 0.0001 |
| 20-39 | 30916(45.21) | 49037(71.71) | 31757(46.44) | | 17937(26.23) | 10955(16.02) | 7132(10.43) | | 5813(8.50) |  |
| 40-59 | 25623(37.47) | 17116(25.03) | 28017(40.97) | | 32263(47.18) | 31689(46.34) | 31094(45.47) | | 28981(42.38) |  |
| 60-80 | 11844(17.32) | 2229(3.26) | 8609(12.59) | | 18183(26.59) | 25739(37.64) | 30150(44.09) | | 33590(49.12) |  |
| BMI(%) |  |  |  | |  |  |  | |  | < 0.0001 |
| <18 | 4335(6.34) | 9218(13.48) | 2995(4.38) | | 1183(1.73) | 465(0.68) | 178(0.26) | | 157(0.23) |  |
| 18-25 | 43280(63.29) | 55828(81.64) | 47923(70.08) | | 34636(50.65) | 24249(35.46) | 20617(30.15) | | 15133(22.13) |  |
| 25-30 | 17424(25.48) | 3221(4.71) | 15749(23.03) | | 27278(39.89) | 34554(50.53) | 37898(55.42) | | 39929(58.39) |  |
| >30 | 3351(4.90) | 116(0.17) | 1716(2.51) | | 5279(7.72) | 9115(13.33) | 9690(14.17) | | 13164(19.25) |  |

Data are presented as mean ± standard error (SE) or percentage.

BMI, body mass index; WC, waist circumference; FBG, fasting blood glucose; OGTT, oral glucose tolerance test; HbA1c, glycosylated hemoglobin; TG, triglyceride; TC, total cholesterol; LDL-C, low density lipoprotein cholesterol; HDL-C, high density lipoprotein cholesterol; SBP, systolic blood pressure; DBP, diastolic blood pressure; UA, uric acid.

**Supplementary Table 2. General characteristics of men**

|  |  | | | Metabolic Syndrome Severity Score | | | |  | | |
| --- | --- | --- | --- | --- | --- | --- | --- | --- | --- | --- |
| Altitude（meters） | Total | 0 | 1 | | 2 | 3 | 4 | | 5 | Pvalue |
| Age(years) | 42.63(0.13) | 34.93(0.24) | 42.44(0.23) | | 45.97(0.34) | 47.66(0.28) | 47.92(0.33) | | 47.25(0.93) | < 0.0001 |
| BMI(Kg/㎡) | 24.58(0.03) | 21.86(0.05) | 23.63(0.04) | | 25.61(0.06) | 27.14(0.06) | 28.44(0.08) | | 28.93(0.20) | < 0.0001 |
| WC(cm) | 86.59(0.08) | 78.21(0.12) | 83.85(0.12) | | 89.63(0.16) | 94.57(0.17) | 98.09(0.18) | | 99.22(0.41) | < 0.0001 |
| FBG(mmol/L) | 5.50(0.01) | 4.85(0.01) | 5.23(0.02) | | 5.65(0.02) | 6.11(0.03) | 6.81(0.05) | | 7.59(0.21) | < 0.0001 |
| OGTT-2h(mmol/L) | 6.50(0.02) | 5.42(0.02) | 5.99(0.03) | | 6.76(0.04) | 7.80(0.07) | 9.05(0.12) | | 9.75(0.34) | < 0.0001 |
| HbA1c | 5.64(0.01) | 5.31(0.01) | 5.48(0.01) | | 5.71(0.02) | 5.99(0.02) | 6.35(0.06) | | 6.71(0.14) | < 0.0001 |
| TG(mmol/L) | 1.79(0.02) | 0.96(0.01) | 1.36(0.01) | | 1.88(0.02) | 2.70(0.07) | 3.60(0.07) | | 5.54(0.27) | < 0.0001 |
| TC(mmol/L) | 4.80(0.01) | 4.38(0.01) | 4.70(0.01) | | 4.94(0.02) | 5.14(0.02) | 5.37(0.03) | | 5.27(0.10) | < 0.0001 |
| LDL(mmol/L) | 2.89(0.01) | 2.58(0.01) | 2.85(0.01) | | 3.03(0.01) | 3.14(0.02) | 3.22(0.02) | | 2.76(0.06) | < 0.0001 |
| HDL(mmol/L) | 1.37(0.00) | 1.49(0.01) | 1.44(0.01) | | 1.33(0.01) | 1.23(0.01) | 1.11(0.01) | | 0.80(0.01) | < 0.0001 |
| SBP(mmHg) | 129.84(0.14) | 116.35(0.17) | 127.77(0.21) | | 135.11(0.30) | 140.03(0.33) | 143.86(0.47) | | 147.24(1.08) | < 0.0001 |
| DBP(mmHg) | 80.71(0.10) | 71.75(0.12) | 79.25(0.15) | | 83.86(0.19) | 87.74(0.22) | 90.89(0.34) | | 93.23(1.02) | < 0.0001 |
| UA(mmol/L) | 362.44(0.69) | 344.66(1.38) | 351.73(1.17) | | 368.46(1.61) | 383.57(1.63) | 396.92(2.52) | | 401.26(6.71) | < 0.0001 |
| Income(%) |  |  |  | |  |  |  | |  | < 0.0001 |
| <30000yuan | 28085(41.07) | 27203(39.78) | 27750(40.58) | | 29678(43.40) | 27846(40.72) | 28133(41.14) | | 24864(36.36) |  |
| ≥30000yuan | 40298(58.93) | 41180(60.22) | 40633(59.42) | | 38705(56.60) | 40537(59.28) | 40250(58.86) | | 43519(63.64) |  |
| Education (%) |  |  |  | |  |  |  | |  | < 0.0001 |
| Senior high school or lower level | 44729(65.41) | 37378(54.66) | 43362(63.41) | | 48832(71.41) | 49605(72.54) | 50781(74.26) | | 52101(76.19) |  |
| College or higher level | 23654(34.59) | 31005(45.34) | 25021(36.59) | | 19551(28.59) | 18778(27.46) | 17602(25.74) | | 16282(23.81) |  |
| Location (%) |  |  |  | |  |  |  | |  | < 0.0001 |
| Urban | 35463(51.86) | 38069(55.67) | 34321(50.19) | | 33959(49.66) | 35949(52.57) | 34704(50.75) | | 34807(50.90) |  |
| Rural | 32920(48.14) | 30314(44.33) | 34062(49.81) | | 34424(50.34) | 32434(47.43) | 33679(49.25) | | 33576(49.10) |  |
| Ethnic(%) |  |  |  | |  |  |  | |  | < 0.0001 |
| Ethnic han | 65326(95.53) | 64328(94.07) | 65135(95.25) | | 65613(95.95) | 66078(96.63) | 66687(97.52) | | 67822(99.18) |  |
| Others | 3057(4.47) | 4055(5.93) | 3248(4.75) | | 2770(4.05) | 2305(3.37) | 1696(2.48) | | 561(0.82) |  |
| Smoke(%) |  |  |  | |  |  |  | |  | < 0.0001 |
| NO | 38746(56.66) | 42568(62.25) | 39922(58.38) | | 37768(55.23) | 34745(50.81) | 33576(49.10) | | 30225(44.20) |  |
| YES | 29637(43.34) | 25815(37.75) | 28461(41.62) | | 30615(44.77) | 33638(49.19) | 34807(50.90) | | 38158(55.80) |  |
| Age(%) |  |  |  | |  |  |  | |  | < 0.0001 |
| 20-39 | 31333(45.82) | 46473(67.96) | 32434(47.43) | | 25274(36.96) | 20659(30.21) | 18217(26.64) | | 19653(28.74) |  |
| 40-59 | 25842(37.79) | 16528(24.17) | 24461(35.77) | | 28523(41.71) | 33508(49.00) | 37529(54.88) | | 36571(53.48) |  |
| 60-80 | 11208(16.39) | 5389(7.88) | 11488(16.80) | | 14586(21.33) | 14217(20.79) | 12637(18.48) | | 12158(17.78) |  |
| BMI(%) |  |  |  | |  |  |  | |  | < 0.0001 |
| <18 | 2646(3.87) | 6982(10.21) | 2387(3.49) | | 834(1.22) | 205(0.30) | 55(0.08) | | 0(0.00) |  |
| 18-25 | 35840(52.41) | 53024(77.54) | 45304(66.25) | | 29104(42.56) | 15995(23.39) | 7283(10.65) | | 4233(6.19) |  |
| 25-30 | 24727(36.16) | 8247(12.06) | 18949(27.71) | | 32400(47.38) | 40626(59.41) | 43191(63.16) | | 43464(63.56) |  |
| >30 | 5163(7.55) | 130(0.19) | 1744(2.55) | | 6045(8.84) | 11557(16.90) | 17862(26.12) | | 20686(30.25) |  |

Data are presented as mean ± standard error (SE) or percentage.

BMI, body mass index; WC, waist circumference; FBG, fasting blood glucose; OGTT, oral glucose tolerance test; HbA1c, glycosylated hemoglobin; TG, triglyceride; TC, total cholesterol; LDL-C, low density lipoprotein cholesterol; HDL-C, high density lipoprotein cholesterol; SBP, systolic blood pressure; DBP, diastolic blood pressure; UA, uric acid.

## Supplementary Table 3. Rate Ratio Calculation of the Five Components at Different MSSSs

|  | **Total(%)** | **MSSS=1(%)** | | **MSSS=2(%)** | | **MSSS=3(%)** | | **MSSS=4(%)** | | **MSSS=5(%)** | |
| --- | --- | --- | --- | --- | --- | --- | --- | --- | --- | --- | --- |
|  | **a** | **b** | **rate ratio** | **b rate ratio** | | **b rate ratio** | | **b rate ratio** | | **b rate ratio** | |
| **WC** | 38.79  34.6/42.98 | 8.13  4.76/11.54 | 20.96  13.76/26.85 | 12.41  11.08/13.76 | 31.99  32.02/32.01 | 11.39  11.52/11.25 | 29.36  33.29/26.17 | 5.66  6.22/5.04 | 14.59  17.98/11.73 | 1.2  1.02/1.39 | 3.09  2.95/3.23 |
| **HDL-C** | 9.27  7.67/10.83 | 1.06  0.51/1.63 | 11.43  6.65/15.05 | 2.07  1.69/2.45 | 22.33  22.03/22.62 | 2.69  2.4/2.95 | 29.02  31.29/27.24 | 2.25  2.05/2.41 | 24.27  26.73/22.25 | 1.2  1.02/1.39 | 12.94  13.3/12.83 |
| **TG** | 28.41  34.97/21.69 | 4.27  5.46/3.06 | 15.03  15.61/14.11 | 8.02  10.54/5.46 | 28.23  30.14/25.17 | 9.25  11.53/6.92 | 32.56  32.97/31.9 | 5.67  6.42/4.86 | 19.96  18.36/22.41 | 1.2  1.02/1.39 | 4.22  2.92/6.41 |
| **FBG** | 25.81  28.67/22.9 | 4.12  4.56/3.68 | 15.96  15.91/16.07 | 7.36  8.79/5.93 | 28.52  30.66/25.9 | 8.25  8.85/7.65 | 31.96  30.87/33.41 | 4.88  5.45/4.25 | 18.91  19.01/18.56 | 1.2  1.02/1.39 | 4.65  3.56/6.07 |
| **SBP** | 40.18  48.06/32.14 | 9.41  12.3/6.48 | 23.42  25.59/20.16 | 13.2  15.86/10.5 | 32.85  33/32.67 | 10.99  12.74/9.21 | 27.35  26.51/28.66 | 5.38  6.14/4.56 | 13.39  12.78/14.19 | 1.2  1.02/1.39 | 2.99  2.12/4.32 |

The total prevalence column(a) calculates the sum of the prevalence of abnormalities in this component for all component permutations, column b calculates the sum of the prevalence of abnormalities in this component under each Metabolic Syndrome Severity Score (MSSS). The rate ratio is calculated as b divided by a.

WC, circumference; HDL-C, high density lipoprotein cholesterol; TG, triglyceride; FBG, fasting blood glucose; SBP, systolic blood pressure.

## Supplementary Table 4. Statistical differences after age stratification for males and females

|  | **Total** | | | | **18-39years old** | | | | | **40-59years old** | | | | **60-80years old** | | | |
| --- | --- | --- | --- | --- | --- | --- | --- | --- | --- | --- | --- | --- | --- | --- | --- | --- | --- |
|  | **Total（%）** | **Men（%）** | **Women（%）** | **Pvalue** | **Total（%）** | **Men（%）** | **Women（%）** | **Pvalue** | **Total（%）** | | **Men（%）** | **Women（%）** | **Pvalue** | **Total（%）** | **Men（%）** | **Women（%）** | **Pvalue** |
| **MSSS** |  |  |  | <0.0001 |  |  |  | < 0.0001 |  | |  |  | <0.0001 |  |  |  | <0.0001 |
| **0** | 18792(35.56) | 7517(29.81) | 11275(41.17) |  | 12415(47.57) | 4776(38.18) | 7639(56.93) |  | 5314(25.08) | | 2100(21.17) | 3214(28.72) |  | 1063(15.53) | 641(19.52) | 422(11.44) |  |
| **1** | 16217(29.40) | 7771(29.90) | 8446(28.90) |  | 7708(28.45) | 3843(29.81) | 3865(27.09) |  | 6576(30.77) | | 2856(29.25) | 3720(32.20) |  | 1933(29.15) | 1072(32.36) | 861(25.85) |  |
| **2** | 11056(19.79) | 5743(22.12) | 5313(17.52) |  | 3985(14.67) | 2394(18.45) | 1591(10.90) |  | 5127(23.69) | | 2441(25.27) | 2686(22.21) |  | 1944(30.21) | 908(28.60) | 1036(31.86) |  |
| **3** | 5915(10.87) | 3294(12.91) | 2621(8.88) |  | 1843(6.94) | 1264(9.80) | 579(4.08) |  | 2920(14.12) | | 1582(16.67) | 1338(11.75) |  | 1152(18.05) | 448(15.00) | 704(21.18) |  |
| **4** | 2016(3.77) | 1152(4.64) | 864(2.92) |  | 560(2.04) | 430(3.25) | 130(0.84) |  | 1054(5.48) | | 581(6.83) | 473(4.21) |  | 402(5.95) | 141(3.94) | 261(8.01) |  |
| **5** | 315(0.62) | 157(0.63) | 158(0.61) |  | 860.33) | 65(0.51) | 21(0.16) |  | 161(0.86) | | 74(0.81) | 87(0.90) |  | 68(1.11) | 18(0.58) | 50(1.65) |  |

The number and proportion distribution of men and women in different age groups under each Metabolic Syndrome Severity Score (MSSS) group. The population was stratified by age as 18-39 years old, 40-59 years old, and 60-80 years old.

# Supplementary Methods

The study was conducted through four stages of random sampling in urban and rural locations in parallel. Developed, developing, and underdeveloped cities were classified based on gross domestic product per capita, concentration of commercial resources, the extent to which a city serves as a commercial hub, vitality of residents, diversity of lifestyle, and future growth potential. At the first stage, one city was selected from each of the 31 provinces in the country. These 31 cities were divided into 10 developed cities, 13 developing cities and 8 undeveloped cities. At the second stage, one district was randomly selected from each city. In the third stage, two communities were randomly sampled from each district. In the final stage, people who met the inclusion criteria and were registered as local residents were randomly selected, stratified by age and sex. The composition of age and sex of each community and urban-rural ratio were decided based on China’s 2010 national census data. A total of 80 937 participants completed the survey and the overall response rate was 92.1%. A parallel random sampling was performed in rural locations. The inclusion criteria were as follow: aged 18 years or older; Having resided locally for at least 5 years; no iodine-containing drugs or contrast agents within 3 months of participation; non-pregnant women. Regarding iodine-containing drugs, included in our questionnaire are amiodarone, potassium iodide, Khumbu, cydiodine buccal tablets, iodine-containing vitamins and others. These drugs are deemed to have little effect on metabolic syndrome components. There are 55 ethnic minorities in China apart from the Han. In the questionnaire, all ethnic minorities except Han were classified as other.
